# Crosstalk between Endothelial Cells and Tumor Cells: A New Era in Prostate Cancer Progression

**DOI:** 10.3390/ijms242316893

**Published:** 2023-11-29

**Authors:** Shiyu Ji, Wenbo Wu, Qi Jiang

**Affiliations:** Department of Urology, Shanghai General Hospital, Shanghai Jiao Tong University School of Medicine, 100 Haining Road, Shanghai 200080, China; yushaoye@sjtu.edu.cn (S.J.); hamstorm@sjtu.edu.cn (W.W.)

**Keywords:** prostate cancer, endothelial cells, tumor microenvironment, therapeutic applications

## Abstract

Prostate cancer stands as one of the most prevalent malignancies afflicting men worldwide. The tumor microenvironment plays a pivotal role in tumor progression, comprising various cell types including endothelial cells, tumor-associated fibroblasts, and macrophages. Recent accumulating evidence underscores the indispensable contribution of endothelial cells to prostate cancer development. Both endothelial cells and tumor cells release a multitude of factors that instigate angiogenesis, metastasis, and even drug resistance in prostate cancer. These factors serve as regulators within the tumor microenvironment and represent potential therapeutic targets for managing prostate cancer. In this review, we provide an overview of the crucial functions of endothelial cells in angiogenesis, metastasis, and drug resistance, and their prospective therapeutic applications in combating this disease.

## 1. Introduction

Prostate cancer (PCa) ranks among the most common malignancies affecting men in Western countries [1]. The primary driver behind PCa development and progression is androgens, making androgen deprivation therapy (ADT) a paramount treatment strategy. ADT encompasses both surgical and pharmacological castration [2]. Despite the initial robust response to treatment due to testosterone level suppression, most patients experience cancer progression within two years, diagnosed as castration-resistant prostate cancer (CRPC) [3]. Recent reports suggest that combining ADT with chemotherapy drugs can enhance survival rates in PCa [4]. However, there remains a subset of patients resistant to chemotherapy [5].

The tumor microenvironment (TME) plays a pivotal role in tumor progression, and is primarily comprised of vascular endothelial cells, cancer-associated fibroblasts (CAFs), tumor-associated macrophages (TAMs), and soluble factors [6]. Previous studies have established a link between TME-derived cytokines and chemokines and PCa pathogenesis [7]. CAFs also wield a significant influence on PCa progression, participating in tumor angiogenesis, metastatic spread, and treatment resistance [8]. Additionally, factors secreted by TAMs have been shown to contribute to proliferation, metastasis, and resistance to therapy [9]. Consequently, the TME in PCa plays a crucial role in tumor angiogenesis, metastasis, and resistance to chemotherapy.

Endothelial cells play a pivotal role in tumor angiogenesis and metastasis [10]. Angiogenesis stands as a fundamental process in tumor progression, where newly formed blood vessels supply vital oxygen and nutrients to the tumor, while simultaneously aiding in waste and carbon dioxide removal, thereby facilitating tumor growth [11]. Furthermore, endothelial cells contribute to tumor metastasis through the secretion of various soluble factors [12]. Equally significant is their involvement in tumor drug resistance [13,14]. As a result, the tumor microenvironment, with particular emphasis on endothelial cells, emerges as a crucial factor in tumor development.

In parallel, tumor cells themselves release an array of soluble factors, including vascular endothelial growth factor (VEGF), which serves a dual role. On the one hand, VEGF promotes the proliferation of vascular endothelial cells, and on the other, it induces tumor cell metastasis by creating a premetastatic niche [15,16,17]. This intricate interplay between endothelial cells and tumor cells actively drives tumor progression.

This review endeavors to delve into the biological significance of endothelial cells within the tumor microenvironment (TME) of prostate cancer (PCa). It centers on the intricate interplay between PCa cells and endothelial cells, specifically elucidating the mechanisms through which endothelial cells contribute to PCa angiogenesis, metastasis, and drug resistance. Additionally, our review examines the potential utility of endothelial cells in prognostication and the treatment of PCa.

## 2. Multiple Factors Derived from Tumor Cells Increase the Proliferation of Endothelial Cells

Tumor angiogenesis is a complex process distinct from angiogenesis in normal tissues. In the latter, blood vessels typically form through sprouting angiogenesis [18], whereas tumor angiogenesis employs multiple mechanisms, including intussusceptive angiogenesis [19], vessel co-option [20], vascular mimicry [21], and sprouting angiogenesis [22]. Angiogenesis results from a delicate balance between pro-angiogenic and anti-angiogenic factors, such as VEGF and thrombospondin-1 (TSP-1). When the pro-angiogenic factors in the stroma outweigh the anti-angiogenic ones, tumor blood vessels begin to form [23]. In normal vascular structures, a layer of pericytes envelops the outer layer of the vascular endothelium. However, in the tumor endothelium, pericyte coverage is significantly reduced, leading to a decreased endothelial cell-to-pericyte ratio. This imbalance between endothelial cell proliferation and insufficient pericyte coverage results in vessel wall instability, subsequently causing tumor bleeding [24]. Maintaining vascular integrity is also dependent on VE-cadherin, which plays a pivotal role. Decreased VE-cadherin levels or the loss of its function disrupts endothelial cell barrier integrity, elevating vascular permeability and facilitating the blood-borne metastasis of tumor cells [25]. Tumor cells often proliferate at a faster rate than tumor angiogenesis can support, resulting in the compression of intra-tumor vessels. Coupled with increased endothelial permeability in tumor vessels, this phenomenon leads to hypoxia in certain tumor regions, subsequently causing tumor hemorrhage and impacting drug delivery [26]. In summary, the tumor vasculature exhibits marked differences from its normal counterpart, characterized by heightened permeability and inadequate perfusion.

Angiogenesis stands as a critical biological process in the growth and metastasis of prostate cancer (PCa) and has emerged as an appealing therapeutic target for castration-resistant prostate cancer (CRPC) [27]. Research has indicated that PSMA-positive membranes secreted by PCa cells possess the ability to induce a pro-angiogenic state in vascular endothelial cells [28]. Meanwhile, the transcription factor Forkhead Box A1 (FOXA1) plays a role in promoting PCa angiogenesis by triggering the expression of various pro-angiogenic factors, including EGF and endothelin-1 [29]. Among the first identified angiogenic growth factors, fibroblast growth factor (FGF) acts on PCa cell FGFR, leading to FRS2α phosphorylation. This, in turn, enhances VEGF-A production through the HIF1α and cJUN pathways, thereby promoting tumor angiogenesis within the microenvironment [30,31].

Tumor-derived transient receptor potential channels (TRP) play a role in various aspects of tumor progression [32]. TRPA1, for instance, facilitates vascular sprouting by regulating Ca^2+^ [33]. Additionally, bradykinin (BK), functioning as an autocrine growth factor, stimulates tumor growth and angiogenesis by prompting the release of FGF and VEGF [34]. Binding to B2 receptors, BK activates Akt, mTOR, NF-κB, and AP-1, ultimately promoting VEGF expression and facilitating angiogenesis in PCa cells [35].

The transition from E-cadherin to N-cadherin, known as “cadherin switching,” promotes the epithelial-to-mesenchymal transition (EMT) process and heightens tumor malignancy [36]. N-cadherin also plays a role in PCa angiogenesis by regulating the expression of monocyte chemoattractant protein-1 (MCP-1) through the PI3k/Akt signaling pathway in PCa cells [37].

The deletion of the chromosome-helix-DNA binding protein 1 (*CHD1*) gene is one of the most common mutations in PCa [38]. *CDH1* deletion increases hypoxia-inducible factor 1α (HIF1α) expression through the downregulation of prolyl hydroxylase domain protein 2 (PHD2), consequently promoting angiogenesis in PCa [39]. HIF1α is a pivotal transcription factor in the tumor angiogenesis process, with the lactylation of HIF1α enhancing the transcription of KIAA1199, further promoting angiogenesis in PCa [40].

N-Myc is involved in the conversion of CRPC to neuroendocrine PCa [41] and also promotes angiogenesis in PCa by mediating TEM8 upregulation [42]. The overexpression of the murine double minute 2 (*MDM2*) gene often leads to p53 inactivation, which promotes tumorigenesis [43]. MDM2 can also enhance PCa angiogenesis by upregulating TNF-α, MMP9, and CXCL10 [44].

Dimethylarginine dimethylaminohydrolase-1 (DDAH1) is frequently upregulated in PCa, and its overexpression leads to the degradation of asymmetric dimethylarginine (ADMA), consequently increasing NO levels. Elevated NO levels, in turn, promote the expression of certain vascular growth factors (e.g., VEGF, HIF1α), ultimately driving tumor angiogenesis [45].

Chronic stress, known to be associated with beta-adrenergic signaling and cardiac hypertrophy [46], is also linked to tumor progression. Beta-adrenergic receptors activate CREB and bind to the promoter of histone deacetylase 2 (HDAC2), inducing its expression. The overexpression of HDAC2, in turn, suppresses TSP-1 expression, thereby promoting PCa angiogenesis [47].

Krüppel-like factor 5 (KLF5) also plays a regulatory role in angiogenesis. The deletion of KLF5 enhances tumor angiogenesis by dampening PI3K/AKT signaling in PTEN-deficient PCa cells, leading to an accumulation of HIF1α [48].

**Figure 1:**
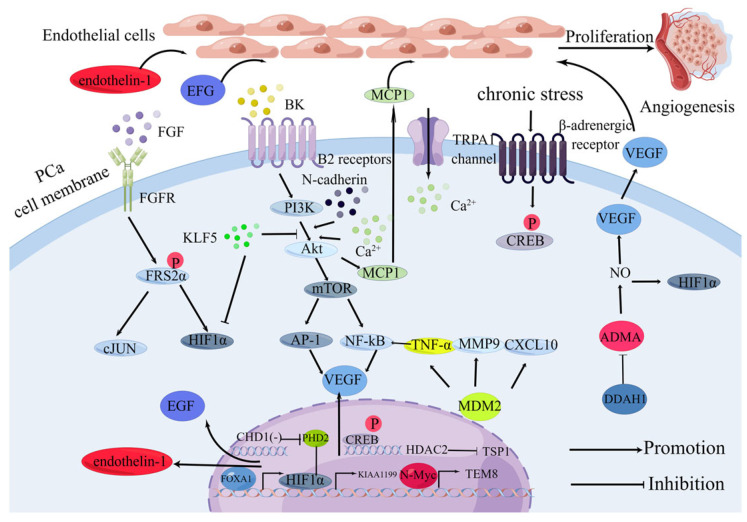
Effect of coding genes on endothelial cells in PCa cells (By Figdraw): FGF: fibroblast growth factor; VEGF: vascular endothelial growth factor; TRPA: tumor-derived transient receptor potential channels A; CHD1: chromosome-helix-DNA binding protein 1; PHD2: prolyl hydroxylase domain protein 2; FOXA1: forkhead box A1; FRS2α: fibroblast growth factor receptor substrate 2 α; BK: Bradykinin; HIF1α: hypoxia inducible factor 1 α; MDM2: murine double minute 2; DDAH1: dimethylarginine dimethylaminohydrolase-1; ADMA: asymmetric dimethylarginine; HDAC2: histone deacetylase 2; KLF5: Krüppel-like factor 5; MCP1: monocyte chemoattractant protein-1. The crosstalk between endothelial cells and tumor cells promotes angiogenesis in PCa. FGF activates endothelial cells through FGF/FGFR/FRS2/cJUN(/HIF1α). BK triggers angiogenesis via BK/PI3K/AKT/mTOR/AP-1(/NF-κB)/VEGF. KLF5 deletion boosts HIF1α via PI3K/AKT. DDAH1 inhibits ADAM, raising NO, enhancing VEGF and HIF1α. MDM2 generates TNF-α, MMP9, CXCL10 for angiogenesis. FOXA1 drives EFG, endothelin-1 for vessel formation. CHD1 deletion increases HIF1α by suppressing PHD2. Lactonization of HIF1α boosts KIAA1199 for angiogenesis. N-Myc upregulates TEM8 for angiogenesis. Chronic stress via β-adrenergic receptors inhibits TSP-1 through CREB/HDAC2 for angiogenesis. TRPA1 regulates Ca^2+^ in angiogenesis. N-cadherin heightens MCP-1 via PI3K/AKT for angiogenesis.

In conclusion, genes encoded in PCa cells stimulate the proliferation of vascular endothelial cells, consequently driving angiogenesis. The effects of these coding genes on endothelial cells in PCa are illustrated in Figure 1.

Simultaneously, the significance of non-coding RNAs in tumor angiogenesis cannot be understated, with miRNAs playing a particularly pivotal role. MiRNAs are a class of short non-coding RNA molecules that regulate target gene expression by specifically binding to the 3′-untranslated region (3′-UTR) of mRNA [49]. However, miRNA expression tends to be downregulated in PCa patients.

For instance, miR-130b can target the TNF-α gene and activate the NF-κB signaling pathway, thereby reducing VEGFA expression and inhibiting angiogenesis in PCa [50]. MiR-185 can target the 3′-UTR of ALK4, inhibiting the nodal/ALK4 signaling pathway and consequently diminishing PCa angiogenesis [51]. MiR-129-5P inhibits angiogenesis in PCa by targeting ZIZ2, which in turn inhibits the Wnt/β-catenin signaling pathway [52]. MiR-195 hinders angiogenesis in PCa by targeting PRR11 expression [53]. MiR-218 suppresses angiogenesis in PCa by targeting the mTOR component RICTOR, leading to a decrease in VEGFA expression [54]. MiR-212 modulates cellular autophagy by targeting SIRT1, thereby restraining PCa angiogenesis [55]. MiR-155 reduces arsenic trioxide-induced angiogenesis in PCa by inhibiting the TGF β/SMAD signaling pathway [56].

In summary, non-coding RNAs, particularly miRNAs, exert inhibitory effects on angiogenesis in PCa by regulating coding genes. However, the decreased expression of miRNAs in PCa patients can lead to opposing results. The influence of non-coding genes on endothelial cells in PCa is detailed in Table 1.

## 3. Interaction between Endothelial Cells and Tumor Cells Induces Metastasis of PCa

Tumor metastasis is a multifaceted process that encompasses several pivotal stages. Initially, tumor cells engage in ECM remodeling, which consists of components such as collagen and elastin. They achieve this by secreting enzymes that enhance their invasive and metastatic capabilities. Notably, matrix metalloproteinases (MMPs) participate in ECM degradation through a process called endocytosis. During endocytosis, the cells frequently undergo epithelial–mesenchymal transition (EMT), a cellular phenomenon marked by the transition from epithelial characteristics to mesenchymal features [57]. EMT is orchestrated by various factors, including tumor cells proliferating in proximity to endothelial and inflammatory cells. These cells release chemokines that attract immune cells and stimulate angiogenesis, collectively contributing to EMT formation [58]. Subsequently, cancer cells infiltrate the bloodstream, embarking on their journey to distant organs, with integrins and E-cadherin playing indispensable roles in facilitating distant metastasis [59,60]. Once in the bloodstream, tumor cells encounter an array of challenges. Circulating tumor cells (CTCs) can disseminate either individually or as clusters. They must contend with issues such as the stiffening of the ECM and the shear forces present in the bloodstream, which collectively constrain their potential for distant metastasis [61]. However, various blood components interact with CTCs to promote distant metastasis. For example, when CTCs interact with platelets, they form a protective coating around cancer cells that aids in evading detection by immune cells [62]. Anoikis resistance among CTCs plays a pivotal role in the distant metastasis of tumor cells. Anoikis is a programmed cell death response triggered when disruptions in the adhesive function of the extracellular matrix occur. Typically, tumor cells manage to evade adhesion-induced cell death, which further propels tumor metastasis [63]. The extravasation of CTCs usually transpires in organs with high vascular permeability, such as the liver and bone [64]. It represents the final step in distant tumor metastasis. Remarkably, metastatic tumor cells often release factors that act at the distant site before metastasis occurs, effectively promoting the spread of tumor cells. This critical process is known as the premetastatic niche (PMN). The PMN plays a pivotal role in tumor metastasis by inducing normal cells in the target organ to recruit the necessary cells. This aids in creating a favorable microenvironment conducive to tumor cell colonization [16]. Similarly, CTCs undergo the process of mesenchymal–to-epithelial transition (MET) before establishing themselves within the parenchyma of distant tissues [65]. To summarize, tumor metastasis is an intricate and multifaceted journey. A more profound comprehension of this process is fundamental for advancing translational treatments.

Distant metastases stand as the primary cause of mortality in PCa patients. Extensive research has underscored the pivotal role of endothelial cells in PCa metastasis. Single-cell sequencing results have further illuminated that activated endothelial cells (aECs) contribute significantly to PCa cell invasion and metastasis [66]. Recent studies have unveiled the influence of CCL5, a chemokine secreted by PCa endothelial cells, on PCa metastasis. CCL5 inhibits androgen receptor (AR) expression in PCa cells, thereby promoting autophagy. Increased autophagy accelerates the disassembly of focal adhesion proteins, ultimately facilitating PCa metastasis [67].

The activation of purinergic P2Y2 receptors (P2Y2R) has been associated with cell adhesion [68]. The endothelial cell activation of P2Y2R induces the secretion of intercellular cell adhesion factor-1 (ICAM-1) and vascular cell adhesion factor-1 (VCAM-1), which enhances the adhesion of tumor cells to endothelial cells, thus mediating PCa metastasis [69].

Additionally, research has shown that the deletion of AKT1 in endothelial cells results in β-catenin phosphorylation and a reduced expression of tight junction proteins such as claudin-5, ZO-1, and ZO-2, promoting PCa metastasis [70].

Connexin (Cx)43 has been found to promote diapedesis in cancer cells. PCa cells with high Cx expression induce an upregulation of endothelial Cx43 through the activation of the intercellular Cx43/ERK1/2/Cx43 axis, facilitating diapedesis in PCa cells [71].

Integrins, on the other hand, inhibit tumor progression. α3β1 integrin, for instance, restrains tumor progression through the α3β1/Abl kinase/Hippo pathway, with reduced α3β1 integrin levels having the opposite effect [72]. Similarly, CXCL16-CXCR6 interactions in PCa promote Ezrin activation and αvβ3 integrin aggregation, leading to MMP expression in PCa cells and thereby enhancing cell migration, invasion, and adhesion to endothelial cells [73].

Furthermore, interleukins play a crucial role in promoting PCa migration. Endothelial cell-secreted interleukin-6 (IL-6) reduces AR expression in PCa cells, subsequently activating the TGF-β/MMP-9 pathway, ultimately leading to PCa metastasis [74]. Insulin and insulin-like growth factor 1 (IGF1) also promote PCa cell adhesion to endothelial cells by enhancing IL-17-induced VCAM-1 expression in endothelial cells [75].

Selectins, particularly E-selectin, have been identified as significant players in tumor metastasis, controlling PCa rolling, adhesion, and metastatic processes [76,77]. Additionally, CCL-2 has been shown to promote tumorigenesis and metastasis in various solid tumors, including PCa [78]. Stat5 has been implicated in reducing E-cadherin expression on the surface of tumor cells, thereby promoting PCa metastasis in both in vivo and in vitro settings [79].

As mentioned above, anoikis resistance plays a role in promoting tumor metastasis [63]. Various mechanisms are linked to the development of anoikis resistance. Syndecan-4 (SDC4) is closely associated with anoikis resistance. Interfering with syndecan-4 expression using miRNA significantly diminishes the adhesion and invasive abilities of tumor cells [80,81]. Moreover, anoikis resistance leads to the remodeling of the extracellular matrix and activation of the PI3K/Akt and Ras/ERK pathways in endothelial cells [82].

The most common site of metastasis in PCa is the bone, and recent studies have revealed that PCa metastasis involves endothelial-to-osteoblast (EC-to-OSB) conversion [83,84]. In metastatic PCa, prostate stem cell antigen (PSCA) is notably overexpressed in metastatic sites, such as the bone, and is associated with a poor prognosis [85]. PSCA overexpression in PCa cells facilitates bone metastasis by interacting with PGRN, upregulating integrin-α4 expression, and activating NF-κB, which, in turn, promotes the adhesion of PCa cells to bone marrow endothelial cells (BMEC).

**Figure 2:**
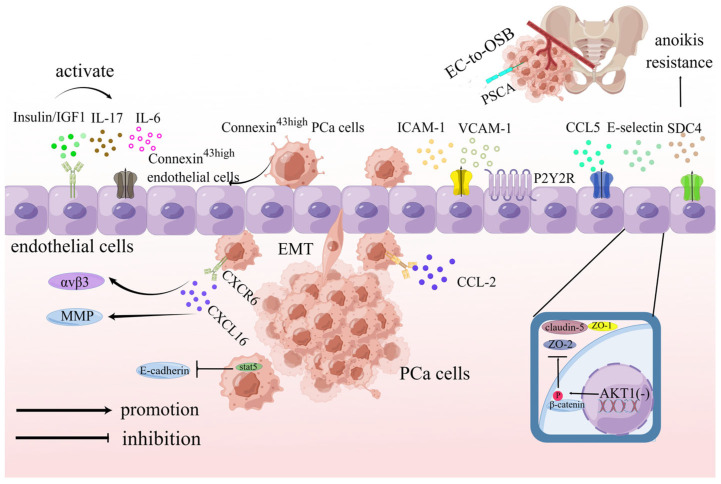
Interaction between endothelial cells and tumor cells induces metastasis of PCa (By Figdraw). IGF1: insulin-like growth factor 1; IL: Interleukin; ICAM-1: intercellular adhesion molecule 1; VCAM-1: vascular cell adhesion molecule 1; CCL: chemokine ligand; SDC4: syndecan 4; P2Y2R: purinergic P2Y2 receptor; MMP: matrix metalloproteinase; CXCR6: chemokine receptor 6; CXCL16: chemokine ligand 16; STAT5: signal transducer and activator of transcription 5; ZO: also known as TJP: tight junction protein. Interaction between endothelial cells and tumor cells induces metastasis of PCa. CCL5 suppresses AR expression, elevates autophagy, promoting PCa metastasis. P2P2R activation enhances PCa adhesion through ICAM-1 and VCAM-1. Loss of AKT1 fosters metastasis by phosphorylating β-catenin, reducing tight junction proteins. Connexin43high cells aid PCa diapedesis. CXCL16–CXCR6 interactions drive metastasis by increasing integrin aggregation and MMP expression. IL-6 reduces AR expression, contributing to PCa metastasis. Insulin and IGF1 amplify IL-17, bolstering PCa cell adhesion. E-selectin and CCL2 promote PCa metastasis. Stat5 downregulates E-cadherin, fueling PCa metastasis. SDC4 facilitates adhesion and invasion through anoikis resistance. PCa triggers bone metastasis via EC-to-OSB conversion and PSCA interaction.

In conclusion, the interplay between endothelial cells and tumor cells plays a pivotal role in driving the distant metastasis of PCa cells. The mechanisms of PCa metastasis are illustrated in Figure 2.

## 4. Endothelial Cells Induced Drug Resistance in PCa and Other Solid Cancers

The role of endothelial cells in promoting angiogenesis and metastasis in PCa has been extensively discussed above. Additionally, endothelial cells exhibit resistance during tumor treatment, including resistance to chemotherapy.

Recent studies have revealed that endothelial cell-secreted FGF2 leads to the upregulation of ETS-related gene (ERG) expression and the activation of the Akt/mTOR signaling pathway in PCa cells, thereby promoting docetaxel resistance [86]. In various solid tumors, endothelial cells have demonstrated the ability to develop drug resistance. For instance, ATP-binding cassette (ABC) transporter proteins are highly expressed in tumor endothelial cells, resulting in chemotherapy resistance [87]. In uroepithelial carcinoma, chemotherapy induces the expression of IL-8 in tumor cells, subsequently upregulating the expression of ABCB-1 in endothelial cells, leading to tumor drug resistance [88].

Soluble factors derived from endothelial cells, such as Notch ligand Jagged1 (Jag1), induce Notch2-Hey1 signaling in lymphoma cells (LCs), contributing to chemoresistance in lymphoma [89]. A loss of endothelial FAK in melanoma/lung carcinoma cells reduces the production of DNA damage-induced cytokines, thus increasing the chemosensitivity of tumor cells to DNA damage treatment in vitro and in vivo [90]. Hepatic endothelial cells play a paracrine role in promoting cell growth and chemoresistance through the activation of HER3-AKT in colorectal cancer cells [91]. In acute myeloid leukemia (AML), the activation of endothelial cells induces the secretion of interleukin-8 (IL-8), or via VEGF-a/VEGFR-2 signaling, leading to resistance to cytarabine (Ara-C) [92,93].

Furthermore, miR-1246 derived from extracellular vesicles of highly metastatic tumor cells induces IL-6 expression, which, in turn, upregulates IL-6 and induces 5-FU resistance through STAT3 and AKT activation in endothelial cells [94]. Tumor endothelial cells with high acetaldehyde dehydrogenase (ALDH) activity have been demonstrated to exhibit drug resistance to 5-FU in melanoma [95]. Additionally, adriamycin (Dox) induces the upregulation of breast cancer resistance protein (ABCG2) and P-GP in endothelial cells, leading to the increased resistance of breast cancer cells to sunitinib [14,96,97]. In patients with multiple myeloma (MM), HIF1α protein in MM endothelial cells may induce angiogenesis and resistance to bortezomib and lenalidomide [98].

In conclusion, factors secreted by endothelial cells can induce drug resistance in PCa and other solid tumors. Further details on drug resistance are provided in Table 2.

## 5. Clinical Perspectives of Endothelial Cells in PCa

### 5.1. Endothelial Cells Can Be Used as Markers for the Treatment and Prognosis of PCa

Previous research has implicated endothelial cells in the diagnosis and prognosis of various diseases, including nephritis [99], gastric cancer [100], pituitary tumors [101,102], and others. Francesca Rivello and her team introduced the Metabolic Assay-Chip (MA-Chip) for the identification and isolation of highly metabolically active cells (hm cells) in the tumor microenvironment (TME). In prostate cancer (PCa) patients, the presence of more than 5 hm cells significantly reduces the probability of survival compared to those with 0 to 5 hm cells, serving as an indicator of poor PCa outcomes [103]. Sebastian Chakrit Bhakdi and colleagues demonstrated that the detection of tumor-associated circulating endothelial cells (tCECs) doubled the positive predictive value (PPV) of independent PSA tests while retaining over 90% of the negative predictive value [104]. Additionally, another study revealed that a high expression of VEGFR1 and NRP1 in endothelial cells predicted the risk of distant recurrence [105]. T. Kosaka and his team found that the density of vasohibin-1 (VASH1) expression in PCa patients correlated with their prognosis. Patients with higher VASH1 density (≥12 per mm) had a 5-year PSA recurrence-free survival rate of 58.8%, while those with lower VASH1 density (<12 per mm) had a rate of 89.1% [106]. C. K. E. Wong and colleagues reported that a high expression of CD31+ and CD45− circulating platelets indicates early recurrence after prostatectomy [107]. Mozhdeh Foroozan and colleagues demonstrated that an elevated expression of the endothelial cell marker CD34 in prostate cancer (PCa) correlates with increased tumor aggressiveness, establishing CD34 as a valuable prognostic indicator [108]. In summary, endothelial cells hold significant clinical relevance as a pivotal marker for assessing PCa progression, thereby aiding in both the treatment and prognosis evaluation of PCa.

### 5.2. Now and the Future: The Role of Endothelial Cells in PCa

The treatment of prostate cancer (PCa) has always presented a significant challenge, especially in the case of metastatic castration-resistant prostate cancer (mCRPC). Classical androgen deprivation therapy (ADT) combined with chemotherapy is a widely accepted approach in the standard management of PCa patients [109]. However, as discussed earlier, endothelial cells can develop resistance to drugs, adding complexity to PCa treatment.

Among the potential drug targets, angiogenesis inhibitors, particularly VEGF antagonists, show great promise. Bevacizumab, the first VEGF inhibitor approved for cancer treatment, has demonstrated its efficacy [110]. A clinical study revealed that PCa patients treated with bevacizumab alongside ADT had significantly improved PSA recurrence-free survival (RFS) compared to those receiving ADT alone [111]. The PI3K/AKT/mTOR signaling pathway is often associated with angiogenesis, making the blockade of this pathway a valuable strategy to inhibit tumor progression [35]. Tesirolimus, an mTOR inhibitor, hinders tumor angiogenesis by disrupting VEGF production [112]. However, a phase II clinical study on temsirolimus monotherapy for CRPC indicated minimal activity in chemotherapy CRPC [113]. Combining two or more chemotherapy drugs can be advantageous for tumor patients. Recent clinical research has shown that the co-administration of two angiogenesis inhibitors (bevacizumab and lenalidomide) alongside docetaxel and prednisone in mCRPC may offer potential clinical benefits [114].

Metastasis represents a bleak clinical outcome in tumor development. While atorvastatin is typically used to enhance endothelial function, some studies have indicated its ability to inhibit the adhesion function of PCa DU-145 cells to endothelial cells [69]. Moreover, the endothelial-to-osteoblast (EC-to-OSB) transition is a common occurrence in PCa bone metastases. Retinoic acid receptor agonists, such as all-trans retinoic acid (ATRA) and palovarotene, can target PCa-induced bone formation to potentially improve the clinical prognosis of patients with bone metastasis [115].

In conclusion, targeting key molecules involved in angiogenesis or metastasis holds promise for effective tumor treatment. Clinical trials involving angiogenesis inhibitors in PCa in recent years are summarized in Table 3.

## 6. Conclusions

In this comprehensive review, we have elucidated the multifaceted role of endothelial cells within the tumor microenvironment (TME). These versatile cells not only play a pivotal role in the angiogenic processes of prostate cancer (PCa), but also significantly contribute to the metastatic cascade. The challenge of drug resistance looms large in the treatment of castration-resistant prostate cancer (CRPC), and the participation of endothelial cells in this resistance phenomenon presents a compelling avenue for future research. Moreover, we have encapsulated the pivotal role of endothelial cells as diagnostic and prognostic markers in PCa management. This underscores their potential utility in early detection, precise diagnosis, and tailored treatment approaches for PCa patients.

To conclude, we have outlined potential therapeutic targets centered around endothelial cells in PCa, with a specific focus on anti-angiogenic agents. Additionally, we have delved into the exploration of recent clinical research endeavors concerning angiogenesis inhibitors in PCa. In essence, these collective insights hold promise for steering PCa prognosis and treatment research into a new and more hopeful direction.

## Figures and Tables

**Table 1:** Effect of non-coding genes on endothelial cells in PCa cells.

MiRNAs	Expression in PCa	Gens and Pathways	Effect on Angiogenesis	Reference
MiR-130b	Down	TNF-α/NF-κB/VEGFA	promotion	[50]
MiR-185	Down	nodal/ALK4	promotion	[51]
MiR-129-5P	Down	ZIZ2	promotion	[52]
MiR-195	Down	PRR11	promotion	[53]
MiR-218	Down	RICTOR	promotion	[54]
MiR-212	Down	SIRT1	promotion	[55]
MiR-155	Down	TGF β/SMAD	promotion	[56]

**Table 2:** Endothelial cells-induced drug resistance in solid cancers.

Key Molecules	Mechanisms of Drug Resistance	Resistant Drugs	Tumor Type	Reference
FGF2	Upregulation of ERG expression and activation of the Akt/mTOR signaling pathway	Docetaxel	PCa	[89]
ABCB-1	The expression of IL-8 and upregulation the expression of ABCB-1	Gemcitabine/cisplatin	Uroepithelial carcinoma	[91]
Jag1	Inducing the expression of Notch2-Hey1 in LCs	Multiple chemotherapy drugs	Lymphoma	[92]
FAK	Loss of FAK reduced the production of DNA damage-induced cytokines	Multiple chemotherapy drugs	Melanoma/lung carcinoma	[93]
HER3	Activation of HER3-AKT	Multiple chemotherapy drugs	Colorectal cancer	[94]
IL-8	Secretion of interleukin-8 (IL-8), or via VEGF-a/VEGFR-2 signaling	Cytarabine	Acute myeloid leukemia	[95,96]
MiR-1246	Inducing IL-6 expression and upregulating IL-6 through STAT3 and AKT activation	5-FU	Melanoma	[97]
ALDH	Activation of ALDH	5-FU	Melanoma	[98]
ABCG2/P-GP	Upregulation of breast cancer resistance protein (ABCG2) and P-GP	Adriamycin (Dox)/sunitinib	Breast cancer	[14,99,100]
HIF1α	Secretion of HIF1α protein	Bortezomib/lenalidomide	Multiple myeloma	[101]

**Table 3:** Angiogenesis inhibitors in clinical trials for the treatment of PCa.

NCT Number	Title	Status	Conditions	Interventions	Characteristics
NCT00795171	Biomarker Study for Sunitinib and Docetaxel in Prostate Cancer	Unknown status	Hormone Refractory PCa	Drug: Docetaxel Sunitinib Drug: Docetaxel	Phase: Phase 2
NCT00684970	Phase IIB Clinical Trial of Hamsa-1™ in Metastatic Castration Resistant Prostate Cancer (CRPC)	Unknown status	Metastatic Castration Resistant PCa (CRPC)	Drug: Hamsa-1™ TL-118	Phase: Phase 2
NCT01683994	Cabozantinib Plus Docetaxel and Prednisone for Advanced PCa	Completed	Prostatic Neoplasms	Drug: Cabozantinib Drug: Docetaxel Drug: Prednisone	Phase: Phase 1 Phase 2
NCT00321646	Neoadjuvant Bevacizumab Plus Docetaxel in High Risk Patients With PCa Undergoing Radical Prostatectomy	Completed	PCa Adenocarcinoma of the Prostate	Drug: Bevacizumab Drug: Docetaxel	Phase: Phase 2
NCT00405574	Study of ATN-224 in Patients With PCa	Unknown status	PCa	Drug: ATN-224	Phase: Phase 2
NCT00631527	Sunitinib Malate, Hormone Ablation and Radiation Therapy in Patients With PCa	Completed	PCa	Drug: Leuprolide Drug: Goserelin Drug: Sunitinib Malate Drug: Casodex Radiation: Radiation Therapy (RT)	Phase: Phase 1
NCT00942578	A Phase 2 Trial of Bevacizumab, Lenalidomide, Docetaxel, and Prednisone (ART-P) for Treatment of Metastatic Castrate-Resistant PCa	Completed	Metastatic PCa	Drug: Bevacizumab Drug: Lenalidomide Drug: Docetaxel Drug: Prednisone	Phase: Phase 2
NCT01083368	Temsirolimus and Bevacizumab in Hormone-Resistant Metastatic PCa That Did Not Respond to Chemotherapy	Completed	PCa	Drug: Temsirolimus Biological: Bevacizumab Genetic: Polymorphism analysis Other: Laboratory biomarker analysis	Phase: Phase 1 Phase 2
NCT00348595	Study of 2 Different Doses of Revlimid in Biochemically Relapse PCa	Completed	PCa	Drug: Revlimid	Phase: Phase 1 Phase 2
NCT00179738	A Multicenter, Single-Arm, Open-Label, Study to Evaluate the Safety and Efficacy of Single-Agent Lenalidomide (Revlimid, CC-5013) in Subjects With Androgen Independent PCa.	Terminated	PCa	Drug: CC5013	Phase: Phase 2

## Data Availability

Not applicable.

## Abbreviations

PCa: Prostate cancer; ADT: Androgen deprivation therapy; CRPC: Castration-resistant prostate cancer; TME: Tumor microenvironment; CAFs: Cancer-associated fibroblasts; TAMs: Tumor-associated macrophages; VEGF: Vascular endothelial growth factor; TSP-1: Thrombospondin-1; FOXA1: Forkhead box A1; FGF: Fibroblast growth factor; TRP: Transient receptor potential channels; BK: Bradykinin; EMT: Epithelial-to-mesenchymal transition; MCP-1: Monocyte chemoattractant protein-1; CHD1: Chromosome-helix-DNA binding protein 1; PHD2: Prolyl hydroxylase domain protein 2; HIF: Hypoxia inducible factor; MDM2: Murine double minute 2; DDAH1: Dimethylarginine dimethylaminohydrolase-1; ADAM: asymmetric dimethylarginine; HDAC2: Histone deacetylase 2; KLF5: Krüppel-like factor 5; ECM: The cytoplasmic matrix; MET: Mesenchymal-to-epithelial transition; PMN: Premetastatic niche; MMP: Matrix metalloproteinases; CTCs: Circulating tumor cells; aECs: Activated endothelial cells; AR: Androgen receptor; P2Y2R: Purinergic P2Y2 receptor; ICAM-1: Intercellular cell adhesion factor 1; VCAM-1: Vascular cell adhesion factor 1; IL: Interleukin; IGF1: Insulin like growth factor 1; SDC4: Syndecan-4; PSCA: Prostate stem cell antigen; BMEC: Bone marrow endothelial cells; ERG: ETS-related gene; ABC: ATP-binding cassette; Jag1: Jagged1; LCs: Lymphoma cells; AML: acute myeloid leukemia; ALDH: Acetaldehyde dehydrogenase; MM: multiple myeloma; MA-chip: Metabolic assay-chip; hm cells: Highly metabolically active cells; tCECs: Tumor-associated circulating endothelial cells; VASH1: Vasohibin-1; mCRPC: Metastatic castration resistant prostate cancer; RFS: Recurrence-free survival; EC-to-OSB: Endothelial to osteoblast; ATRA: All-trans retinoic acid.
